# Advances in Engineering Circular RNA Vaccines

**DOI:** 10.3390/pathogens13080692

**Published:** 2024-08-15

**Authors:** Zhongyan Zhang, Yuanlei Fu, Xiaoli Ju, Furong Zhang, Peng Zhang, Meilin He

**Affiliations:** 1 School of Pharmacy, Yantai University, Yantai 264005, China; zzy18357805668@163.com; 2 Yantai Key Laboratory of Nanomedicine & Advanced Preparations, Yantai Institute of Materia Medica, Yantai 264005, China; ylfu@simmyt.ac.cn (Y.F.); xlju@simmyt.ac.cn (X.J.); frzhang@simmyt.ac.cn (F.Z.)

**Keywords:** circular RNA vaccines, circularization, RNA therapeutics, mRNA, RNA delivery

## Abstract

Engineered circular RNAs (circRNAs) are a class of single-stranded RNAs with head-to-tail covalently linked structures that integrate open reading frames (ORFs) and internal ribosome entry sites (IRESs) with the function of coding and expressing proteins. Compared to mRNA vaccines, circRNA vaccines offer a more improved method that is safe, stable, and simple to manufacture. With the rapid revelation of the biological functions of circRNA and the success of Severe Acute Respiratory Coronavirus Type II (SARS-CoV-2) mRNA vaccines, biopharmaceutical companies and researchers around the globe are attempting to develop more stable circRNA vaccines for illness prevention and treatment. Nevertheless, research on circRNA vaccines is still in its infancy, and more work and assessment are needed for their synthesis, delivery, and use. In this review, based on the current understanding of the molecular biological properties and immunotherapeutic mechanisms of circRNA, we summarize the current preparation methods of circRNA vaccines, including design, synthesis, purification, and identification. We discuss their delivery strategies and summarize the challenges facing the clinical application of circRNAs to provide references for circRNA vaccine-related research.

## 1. Introduction

In recent years, mRNA vaccines have been developed as a result of the SARS-CoV-2 epidemic, and mRNA vaccines containing antigenic sequences have been rapidly developed in research and clinical practice [1]. Circular RNA (circRNA) is a novel RNA mode for disease therapy that is now being extensively studied. In 1976, Sanger et al. discovered a single-stranded closed-loop RNA molecule in plant-like viruses, and circRNAs were thus recognized [2]. Since 2010, the research on circRNA has exploded with advances in RNA sequencing technology. Traditionally, circRNA has been categorized as a non-coding RNA [3]. It is a covalently closed-loop structure without a 5′ end cap and 3′ end tail structure. As a result, it is highly resistant to RNA exonucleases, which are more stable in tissues or body fluids than linear transcripts of the same gene. The biological function of endogenous circRNAs is to regulate gene expression and thereby regulate disease progression, making them potential disease biomarkers [4,5,6]. In 2017, a study showed that circRNA could be extensively translated, and as a result, it was recognized that circRNA is able to encode and express proteins [7]. In 2018, researchers successfully synthesized circRNA capable of expressing proteins [8]. Furthermore, since 2022, the application of synthetic circRNA to express pertinent antigens and thus elicit adaptive immune responses has demonstrated beneficial and preventive outcomes in the treatment of malignant melanoma that is difficult to treat, as well as coronavirus (COVID-19) [9]. Based on the current understanding of the molecular biological properties and immunotherapeutic mechanisms of circRNA, this paper systematically reviews the in vitro synthesis techniques and delivery strategies of circRNA vaccines and identifies the key issues and challenges facing the clinical transformation of circRNA vaccines, providing insights and perspectives for future research.

## 2. The Biological Functions of Natural circRNA

Natural circRNAs have been widely found in multiple species, ranging from viruses to mammals, and can be categorized according to their composition: exon circRNA, intron circRNA, or exon-intron circRNA. Exon-derived circRNA is the most abundant and widely studied type, mainly distributed in cytoplasm [10]. Unlike the formation of conventional linear RNA, circRNA comprises novel RNA molecules produced by reverse splicing the exons or introns of precursor mRNA. During circRNA biogenesis, the 3′ end of the downstream exon attacks the 5′ end of the upstream exon, creating covalently bound 5′-3′ tails and loops [11]. Natural circRNA performs a range of coding and non-coding functions within the cell. CircRNAs can act as miRNA sponges to regulate miRNA expression. Among them, the most representative circRNA is ciRS-7. ciRS-7 is an antisense chain of the transcription of cerebellar degeneration-related protein 1 (CDR1as), which contains more than 70 conserved binding sites for miR-7 [12]. It can act as a molecular sponge for miR7 and inhibit the activity of miR-7, thus promoting the expression of miR-7 target proteins to regulate the course of disease [13,14]. In addition, it has been reported that circRNA can encode proteins [15,16]. CircRNA was first found to encode and translate proteins in Drosophila in 2015, and this coding function provided a theoretical basis for the development of circRNA vaccines [17]. Due to the lack of a 5′-cap and a 3′-tail, circRNAs do not rely on the 5′ cap to initiate translation, but are translated in vivo or in vitro via N6-methyladenosine (m6A) modification or by containing an internal ribosome entry site (IRES) [18,19]. There are a large number of m6A motifs on circRNAs in human cells, and a single m6A site can initiate the translation of circRNA with the involvement of multiple proteins [7]. According to Chen et al., the methyltransferase METTL3 catalyzes the translation of m6A-methylated CircMAP3K4 into CircMAP3K4-455aa, which is dependent on the mRNA-binding protein IGF2BP1. This protein interacts with apoptosis-inducing factor (AIF) to protect it from cleavage and reduce its nuclear distribution, ultimately leading to cisplatin resistance in liver cancer cells [20]. IRES is an RNA element that recruits ribosomes to internal regions of RNA to initiate translation and can recruit different trans-acting factors to facilitate ribosome assembly and initiate translation [21]. circ-ZNF609 [22,23] and circFGFR1 [24] have been shown to be accessible for translation using IRES, and m6A modification could enhance the efficiency of the IRES-mediated translation of circZNF609. In addition, circRNAs with multiple open reading frames (ORFs) but without stop codons translate proteins in a the rolled-over amplification manner [25]. These studies laid the foundation for the application of engineered circRNA in vivo.

## 3. Molecular Biology of circRNA Vaccines

### 3.1. Characterization of circRNA Vaccines

The most commonly used vaccines are based on pathogens, DNA, and peptides, each with different characteristics and limitations (Table 1). For example, mRNA vaccines are quickly broken down by nucleic acid exonucleases and have a brief half-life. DNA vaccines have genomic integration defects [26]. CircRNA vaccines have no risk of genomic integration and can be processed in the cytoplasm to produce antigens. Because of their covalently closed structure and lack of termini, they are not affected by exonuclease mediated degradation, and thus maintain higher stability than the linear mRNA subtype [11,27]. It has been shown that engineering circRNAs can improve their translational efficiency, produce more proteins in vitro than mRNA, and exhibit higher translational persistence both in vitro and vivo [28,29]. Delivery options for circRNA vaccines include encapsulation in a delivery vehicle like lipid nanoparticles (LNPs) or distribution in a naked form. CircRNA-LNP show higher thermal stability than mRNA-LNP vaccines. According to literature reports, circRNA vaccines encapsulated in LNPs are stored at 4 °C for no less than 4 weeks, while mRNA vaccines are only stored for up to 3 weeks [30]. In addition, it has been shown that engineered circRNAs can be recognized by the natural immune receptor retinoic acid-inducible gene protein-I (RIG-I), which triggers the intracellular natural immune response and inhibits infection, suggesting that circRNA can induce an innate immune response and is immunogenic [31,32]. At present, the limitations and shortcomings of circRNA vaccines are still unclear, and their safety issues still need to be further studied. The technology for producing circRNA vaccines is not yet mature enough for large-scale production.

### 3.2. Mechanism of Immunotherapy Mediated by circRNA Vaccines

CricRNA vaccines containing specific encoding antigens reach the cytoplasm of host cells through the delivery system, and then are translated into protein antigens through the ribosome to cause antigen-specific immune response and produce the RNA self-adjuvant effect, which increases the host’s resistance to viruses and malignancies [44]. The expressed antigens are presented to CD8^+^ T cells by major histocompatibility complex (MHC) class I molecules to activate cellular immunity. In addition, some antigens are recognized by MHC II and presented to CD4^+^ T lymphocytes, further activating cellular and humoral immunity. CircRNA also increases the expression of co-stimulatory molecules and the secretion of several inflammatory cytokines and chemokines by inducing the maturation of dendritic cells. For instance, Yang et al. stated that the intratumoral administration of a circRNA mixture encoding four cytokines (including interleukin-15 (IL-15), interleukin-12 (IL-12), granulocyte–macrophage colony-stimulating factor (GM-CSF), and interferon-α2b (IFN-α2b)) can lead to the activation of T cells (CD4^+^ and CD8^+^ T cells) and promote the infiltration of immune cells, thus exerting a powerful anti-tumor effect [45]. In a study by Qu et al., a circRNA-RBD vaccine expressing spiny protein trimer (RBD) elicited a significant Th1-biased immune response and produced a high proportion of neutralizing antibodies, which activated the specific immune response. Meantime, in this study, we found that due to the immunogenicity of circRNA, it can be used as a self-adjuvanted vaccine. The circRNA-RBD vaccine can induce a significant expression of IL-6 and monocyte chemoattractant protein-1 (MCP-1), suggesting the activation of the innate immune response [30].

### 3.3. Enzymatic Degradation Mechanisms of circRNA

CircRNA, a closed-loop structure, has no free ends that protect it from exonuclease cleavage. Therefore, circRNA is less likely to be degraded by conventional linear RNA degradation pathways. However, RNA-endonuclease may linearize circRNA and cause degradation. CircRNA is resistant to RNase R cleavage in the short term. The use of RNase H and Rrp44 in vitro showed the first signs of circRNA degradation by endonucleases, but with low efficiency [46]. Subsequent studies found that double-stranded RNA (dsRNA) and polyinosine–polycytidic acid (poly (I:C)) can activate the endonuclease RNase L in vivo, which leads to the global degradation of circRNA [47]. At present, the miRNA-mediated degradation mechanism is the most well-characterized circRNA degradation pathway. For example, ciRS-7, also known as CDR1as, can be degraded by a highly complementary binding site of a specific miRNA: miR-671 [48]. This miRNA binding site can trigger the cleavage of the resulting RNA double-stranded by Argonaute 2 (AGO2), a key component of the RNA-induced silencing complex [31]. Nevertheless, the mechanism of circRNA degradation in vivo is still not fully elucidated. As circRNA shows excellent potential for RNA therapy, its degradation mechanism should be further studied in the future to ensure its high stability as a therapeutic agent in vivo.

## 4. In Vitro Synthesis Process of circRNA

mRNAs are not suitable for rapid and economic mass production due to their instability, high production costs, and complex manufacturing processes [49,50]. In contrast, circRNA is more stable and easier to store. Usually, the in vitro synthesis process of circRNA includes the design and synthesis of linear precursors, circularization, purification, and identification.

### 4.1. Design and Synthesis of Linear RNA Precursors

The design of linear RNA precursors for circularization must incorporate several components that contribute to the overall properties and functionality of the vaccine. Linear RNA precursor must be designed to include the ORF region and the translation-mediating IRES element, a specialized sequence located upstream of the start codon that binds to the 40S ribosomal subunit and induces downstream reading and translation in a 5′ cap-independent manner [51,52,53]. The downstream ORF elements of IRES can store encoded information, and the 40S ribosome subunit recognizes and binds to the start codon of the ORF and ends at the end codon of the ORF during translation. The commonly used IRESs are mainly derived from encephalomyocarditis virus (EMCV), coxsackievirus B3 (CVB3) [54], human rhinovirus B3 (HRV-B3), poliovirus 1 (PV1), hepatitis C virus (HCV), etc. [30]. The translational efficiency of circRNAs is critical for their application as therapeutic agents or vaccines, and thus their constituent components need to be rationally optimized. ORF fragment sizes are typically above 1000 nucleotides (nt), and there may be a requirement to express binding or fusion antigens. Taken together, this significantly increases the length of linear precursor mRNAs. Moreover, longer circRNAs are more easily sheared and more prone to mutation [55]. The length of commonly used IRESs such as EMCV or CVB3 is about 700 nt, and the circRNA length of even the least translated proteins is close to 1000 nt, which raises the pressure for cyclization and the development of secondary structures [8]. Therefore, shorter IRES analogs, such as Kozak sequences and AU-rich sequences, need to be screened in the future to improve translation efficiency [25]. RNA-binding proteins (RBPs) can be attracted to the 5′ and 3′ untranslated regions (UTRs) of mRNA in order to start translation [56]. Although circRNA does not have any UTRs, motif-specific UTR analogs can be rationally designed in the areas upstream and downstream of the linear RNA precursor IRES-ORF box to mimic RBP binding sites and thereby improve translation efficiency. For example, upstream PABP motifs and downstream HBA1 motifs can significantly improve translation efficiency [28]. In addition, the larger the fragment between the 5′ and 3′ splice sites of a linear RNA precursor containing complex elements, the lower its splicing efficiency, which can facilitate its cyclization and further improve translation efficiency by adding homology arms and unstructured spacer sequences.

Typically, linear RNA precursors can be synthesized chemically or by in vitro transcription (IVT) enzymatic processes [57,58]. The chemical synthesis technique uses nucleoside phosphoramidite monomers as the basic component of linear oligonucleotide synthesis. However, the corresponding synthesizer equipment can usually synthesize less than 70–80 nucleotides of small RNA [59]. The IVT enzymatic process is performed using three primary components: DNA-dependent RNA polymerase, ribonucleotide triphosphate, and a double-stranded DNA template [45]. Typically, RNA polymerases utilize phage RNA polymerases, like T7 or SP6 polymerases [60]. A large number of linear precursors are produced by amplifying the DNA template’s target sequence. This is simpler and faster than large-scale techniques for purifying and producing proteins. Chen et al. claimed that long-lasting protein synthesis in vivo can be achieved by optimizing circRNA elements like the 5′ and 3′ UTRs, carrier architecture, and IRESs. These modifications can boost protein production by hundreds of times [28].

### 4.2. Circularization

The key process step of circRNA synthesis in vitro is the cyclization of linear RNA molecules using chemical or enzymatic means [61]. With the increasing depth of circRNA-related research, three main cyclization methods have emerged: chemical ligation, enzymatic ligation, and the ribozyme method.

#### 4.2.1. Chemical Synthesis

**Figure 1:**
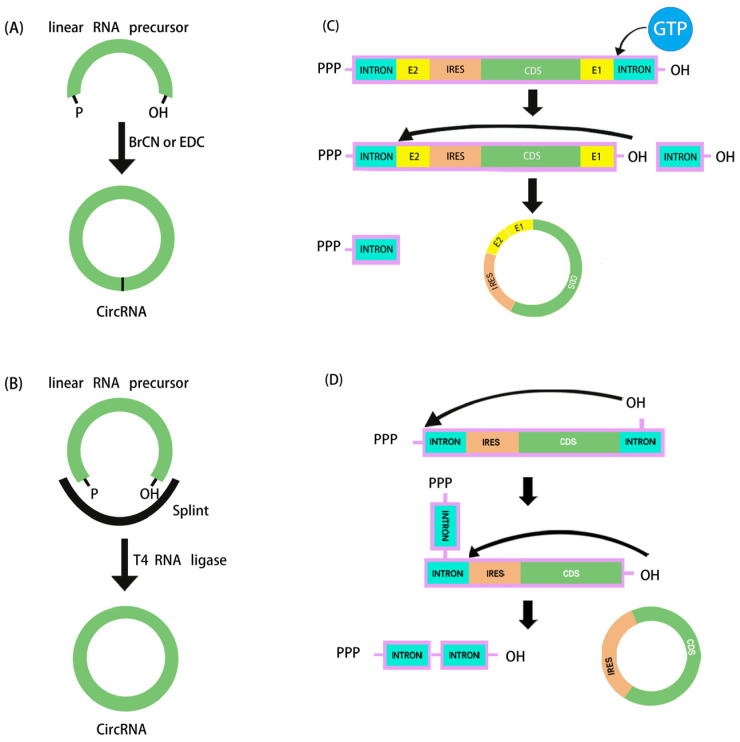
Schematic diagram of in vitro artificial RNA cyclization methods. (**A**) Chemical synthesis. (**B**) Ligation by T4 RNA ligase. (**C**,**D**) The ribozymatic process used in in vitro cyclization to synthesize circRNA: (**C**) cyclization based on type I intron PIE system, (**D**) cyclization based on type II intron self-splicing.

The chemical method for synthesizing circRNA originated in 1988, and involves using cyanogen bromide (BrCN) or 1-ethyl-3-(3-dimethylaminopropyl) carbodiimide (EDC) to form a covalent bond between the 5′-terminal phosphoryl group and the 3′-terminal hydroxyl group of linear RNA (Figure 1A). However, side reactions may occur during this cyclization reaction, producing circRNA variants that contain a 2′-5′ phosphodiester bond at the junction of the linear molecules [62]. With longer RNA fragments it would be difficult to connect the two ends. Considering the exorbitant price, poor yield, and that it is only suitable for the cyclization of RNAs up to 70 nucleotides in length, this method is no longer widely used [59]. In recent years, the application of click chemistry has overcome the inherent shortcomings of the chemical synthesis method, with the advantages of fast reaction kinetics, quantitative yield, few by-products, high chemical specificity, biocompatibility, and regioselectivity. In the future, further in vivo experiments are needed to determine whether it is suitable for widespread use [63].

#### 4.2.2. Enzymatic Strategies

Enzyme-based ligation methods are commonly used for the preparation of circRNA. These ligases include T4 DNA ligase (T4 Dnl 1), T4 RNA ligase I (T4 Rnl 1), and T4 RNA ligase II (T4 Rnl 2), which are produced from E. coli infected with T4 phage. Double-stranded DNA and DNA/RNA hybrid strands can be ligated by T4 Dnl 1. The cyclization of RNA usually uses single-stranded RNA as the substrate, so a DNA template complementarily paired with the sequences at the ends of the RNA nick needs to be designed as a splint sequence to achieve cyclization. The advantage of this method is the higher accuracy of ligation, but it requires a linear RNA precursor with no obvious RNA secondary structure at the junction. Due to inefficient cyclization and the requirement for a large amount of enzymes, only a few studies have used T4 Dnl 1 for the cyclization of RNA [31]. Through three nucleotide transfer stages and an ATP-dependent mechanism, T4 Rnl catalyzes the creation of covalent 3′,5′-phosphodiester linkages between the 5′-phosphate and 3′-hydroxy terminal groups of linear RNA precursors (Figure 1B). circRNA catalyzed by T4 Rnl shows minimal immunogenicity without the need to introduce exogenous sequences. Though it is only appropriate for linear RNA precursors less than 500 nt without secondary structures at the terminal ends, T4 Rnl 1 may accomplish effective single-stranded RNA binding [64]. In addition, due to the low reaction specificity of T4 Rnl 1, intermolecular ligation can form oligomeric by-products. To improve cyclization efficiency, partially complementary RNA splint sequences need to be designed to bring the two ends close together while maintaining a single-stranded state 2–3 nucleotides from both ends to allow ligation via T4 Rnl 1 [65]. In order to synthesize IVT circRNA up to 4 kb in length, Carmona et al. developed an optimal motif for the formation of an RNA hairpin structure that effectively brings the 3′ and 5′ ends closer together [66].

#### 4.2.3. Ribozymatic Methods

The ribozymatic method is the most commonly used method of RNA cyclization and is particularly applied in the manufacture of large fragments of circRNA. Type I introns and type II introns can perform RNase functions that enable linear RNA molecules to self-splice to form circRNA without the need for other enzymes. Currently, the most widely studied is the use of type I introns, also known as the permuted intron–exon (PIE) method. Using type I introns derived from the Anabaena pre-tRNAleu gene, the PIE technique was initially applied in 1992 to produce circRNA [67]. Two years later, with the identification of the type I intron of the thymidylate synthase (Td) gene from T4 phage, the Td exon was used to synthesize circRNA [68]. To facilitate cyclization in the PIE method, the reaction system merely has to be supplemented with GTP and Mg^2+^ (Figure 1C).

In 2018, based on the PIE method, Alexander Wesselhoeft et al. realized effective cyclization RNA up to 5 kb by reasonably designing auxiliary splicing sequences that were translated in eukaryotic cells for the first time. Moreover, it was demonstrated that the half-life of proteins expressed by the engineered circRNA cells in vitro could be up to 116 h, whereas it was only 49 h for the corresponding linear mRNA modified with pseudouridine and 5-methylcytosine [8]. By adopting the PIE approach, Wei et al. recently developed a circRNA vaccine encoding the trimeric receptor-binding domain (RBD) antigens of the SARS-CoV-2 spike. The vaccine elicited a high proportion of neutralizing antibodies and T-cell responses in rhesus monkeys and provided effective protection against the Delta and Omicron mutants [30]. On the other hand, at the terminal junction location of circRNA, type I intronic self-splicing produces an RNA segment (E1 and E2, referred to as the scar sequence) greater than 80 nt, which may cause significant immunogenicity in vivo [69]. Type II introns, as opposed to type I introns, can be utilized to create circRNA cyclization without the need for leftover exogenous sequences (Figure 1D), but the exact mechanism and wide applicability of this method still need to be verified by more studies. In order to solve the problem of scar sequences, the CureMed team developed a method called Clean-PIE, which astutely identifies the ideal looping site by examining the IRESs or protein-coding sections [70]. This cyclization method does not introduce exogenous sequences. Therefore, it can be used to produce scarless circRNA with minimal immunogenicity. In addition, Zefeng Wang’s team developed a novel method called CirCode, which uses type II intron self-splicing in a looping manner of co-transcription [55]. This method can efficiently generate scarless circRNA with low immunogenicity and can be produced on a large scale. Synthesized circRNA is very stable and can prolong the time of protein translation in different cells.

### 4.3. Purification and Identification of circRNA Vaccines

The preparation process of circRNA vaccines mainly includes the synthesis of linear RNA precursors by in vitro transcription using plasmid as a template, cyclization to form circRNA, and the encapsulation of the vaccine by LNPs and other delivery systems. Therefore, it is crucial to obtain high-purity plasmids by fermentation, pyrolysis, and purification. Subsequently, linearized plasmids need to be obtained by restriction endonuclease and purified by chromatography to remove enzymes and salts from the reaction system. Linear RNA precursors are synthesized using linearized plasmids as templates, and their yield and purity can be improved by optimizing transcriptional reaction conditions. Finally, the purification of linearized RNA precursors is usually achieved by ultrafiltration to remove excess nucleotides and salts, and the DNA template in the reaction system is removed by the DNase I enzyme prior to purification.

The by-products of the intron-splicing cyclization reaction include unsuccessfully cyclized linear RNA precursors, spliced RNA intron fragments, nucleotide triphosphates, and open-loop RNA (nicked-circRNA). Because linear RNA precursors and open-loop RNAs have similar molecular weights to the corresponding circRNA, the removal of linear RNA impurities is a challenge in the production of circRNA vaccines. It has been reported that high-performance liquid chromatography (HPLC) or enzyme digestion by RNase R can be used to remove linear RNA precursors and other impurities [55]. However, low doses of RNase R might not be able to sufficiently degrade linear RNA, but high amounts of RNase R can also cause circRNA breakdown. In order to optimize enzyme digestion by RNase R, the incubation period and reaction conditions must be adjusted. [71]. In addition, RNase R is not yet available for mass production due to its high production cost. These results illustrate the urgency of exploring better methods to improve the purification approach, as there are multiple drawbacks in circRNA purification with RNase R. Size-exclusion chromatography (SEC) can separate compounds based on molecular weight and is more suitable for removing smaller intron fragments. SEC methods generally use longer columns and relatively low flow rates. Thus, the resolution is very limited for compounds with small molecular weight differences. Combining SEC-HPLC and RNase R digestion is currently the most efficient purification technique to widen the property gap of compounds between circular RNA, open-loop RNA, and linear RNA precursors so as to further improve purification and separation efficiency. The final circRNA product should be highly purified and endotoxins should be removed by ethanol precipitation or RNA gel electrophoresis [65].

After that, the structure of the synthetic circRNA should be identified. The junction site of circRNA is a head-to-tail connection provided by the two flanks of the linear RNA precursor. Therefore, it is only in the circRNA and not in the linear RNA precursor. After reverse transcription and cDNA synthesis, the existence of the circRNA can be determined by PCR using a pair of primers covering the region that contains the junction point [8]. Furthermore, identifying the molecular dimensions and sequencing of the aforementioned PCR products can help ascertain whether the circRNA synthesis was successful [72]. The accuracy and sequence characteristics of circRNA can be verified by sequencing [73].

## 5. Delivery Strategies for circRNA Vaccines

**Figure 2:**
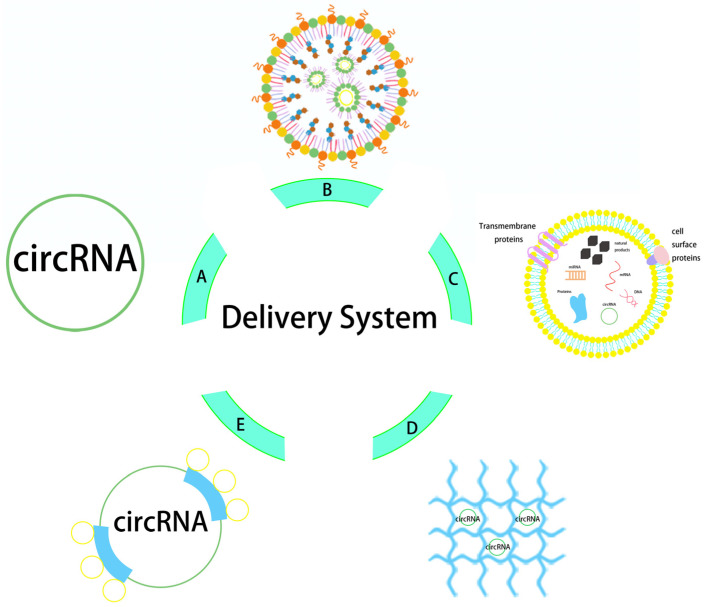
Structure diagram of circRNA delivery system. The delivery vectors from (**A**–**E**) are naked circRNA direct delivery, LNP, exosome, hydrogel, and protamine.

In order to properly protect engineered circRNA vaccines and transfer them across membranes into cells, delivery mechanisms are essential. The electronegativity and hydrophilicity of nucleic acid drugs are the main obstacles to crossing the anionic lipid bilayer of the cell membrane [74,75]. In addition, delivery systems can protect nucleic acid drugs from degradation by endogenous nuclease and the surveillance of the autoimmune system, which helps deliver them to their target sites for expression [76]. Recent reports on the delivery modes for engineered circRNA mainly include carrier-free delivery, lipid nanoparticle delivery, exosome delivery, and other potential carrier delivery methods (Figure 2).

### 5.1. Carrier-Free Delivery

The unique covalent closed-loop structural features of circRNAs facilitate their delivery to tissues, cells, and subcellular organelles. Their terminal-free structure helps to resist exonuclease degradation and is suitable for carrier-free drug delivery. For instance, Yang and colleagues dissolved naked circRNA encoding luciferase in PBS and injected it directly into four mouse models of B16F10 melanoma, MC38 colon cancer, A549, and NCI-H358 non-small cell lung cancer. After 6 h of intratumoral injection, significant bioluminescence was specifically detected in all four tumor models. In parallel, this study also found that circRNA dissolved in Ringer’s solution could induce higher luciferase expression at the tumor site than that dissolved in PBS. And further verification showed that it was mainly affected by CaCl_2_ and KCl in Ringer’s solution. This suggests that the intratumoral delivery efficiency of naked circRNA can be improved by adjusting the solvent composition of circRNA [45]. Because it is more stable than mRNA and has a lower immunogenicity, circRNA can be injected directly. Thus, more research on naked circRNA vaccines in vivo and in clinical trials is required. Remarkably, research has demonstrated that traditional physical methods like electroporation, gene guns, and microneedles can enhance the effectiveness of naked mRNA antigen presentation [77]. Husseini et al. achieved the intradermal delivery of a naked mRNA vaccine encoding the tumor-associated antigen human gp10025-33 via ion electroporation (IP). The vaccine induced a robust immune response in a mouse model of melanoma, leading to tumor suppression and the up-regulation of cytokine expression [78]. This implies that naked circRNA vaccine administration could be facilitated by the same strategy.

### 5.2. Lipid Nanoparticle Delivery

Liposomes, the pioneer of nano-medicine delivery systems, are bilayer vesicle structures formed by lipid molecules. They have both a hydrophobic outer layer and an internal aqueous phase cavity that can carry both hydrophobic and hydrophilic drugs [79]. Smaller-sized liposomes are able to escape phagocytosis and enter the target cells [80]. Therefore, LNPs with a size of around 100 nm are more suitable for the targeted delivery of nucleic acid drugs. They are also biocompatible, easy to shape, and have a high payload capacity [81]. At present, an siRNA-LNP drug (Onpattro) and two mRNA-LNP novel coronavirus SARS-CoV-2 prophylactic vaccines (BTN162b2 [82] and mRNA-1273 [83]) have been approved for marketing by the FDA. LNPs consist of four main components: cationic ionizable lipids, neutral phospholipids, cholesterol used to stabilize LNP membranes, and polyethylene glycol-modified lipids that affect particle size and colloidal stability [84]. Cationic lipids are the key components of LNPs [85,86], and are able to bind to negatively charged RNA to improve encapsulation efficiency and transfection efficiency [87]. During the delivery process, LNPs are mainly taken up by endocytosis after fusion with the cell membrane and then form endosomes in the cytoplasm [88]. A non-bilayer structure is formed when the cationic lipids in LNPs and the anionic lipids in endosomes bond. Then, RNA is released to initiate the translation process after the endosomal membrane is unwound [89,90].

Recently, a variety of novel cation-ionized LNP strategies for circRNA delivery have been reported (Table 2). For example, the purified hEpo circRNA encapsulated in LNPs was highly expressed in 293T cells, which demonstrated the potency of the in vivo delivery of circRNA through LNPs [91]. A recent study showed that circRNA encoding OVA-luciferase (circRNAOVA-luc) can form a stable spherical complex using LNPs with a size of about 74 nm. It generates immune responses and activates antigen-specific T cells in mouse models. Moreover, anti-tumor efficacy was confirmed in three mouse tumor models after vaccination with circRNAOVA-luc-LNP. It was clear that tumor growth was significantly inhibited, and circRNA showed better efficacy and protein expression compared to linear RNA. This finding not only confirms that LNPs can be used as a delivery system for circRNA vaccines, but also suggests that circRNA may be a better choice for neoantigen vaccines [9]. Li et al. used a high-throughput combinatorial approach to synthesize and screen H1L1A1B3 LNPs that can efficiently deliver circRNA to lung tumors. The efficiency of H1L1A1B3 LNP to deliver circRNA to lung cancer cells is 4-fold higher than that of the LNPs (ALC-0315) used by Pfizer/BioNTech in the mRNA COVID-19 vaccine, and it also has a powerful immune-activation effect. The H1L1A1B3 LNPs that delivered circRNA encoding IL-12 elicited potent immune responses leading to marked tumor regression in a mouse model of lung cancer after a single tumor injection or intratracheal administration [92]. In addition, IL-12 circRNA LNPs also exhibited higher stability and sustained IL-12 expression compared to mRNA. These results highlight the potential of the LNP platform to advance RNA delivery in cancer therapy and broaden the prospects of RNA immunotherapy.

Despite the fact that LNPs are presently the most popular RNA vaccine delivery method, they still face great challenges. First, their poor heat resistance leads to the need for extremely low temperatures for the transport and storage of such vaccines. Second, due to various administration methods, the inflammatory nature of LNP components may result in negative effects or even death [93]. In order to successfully overcome these limitations, recent studies have concentrated on creating lyophilized LNP formulations and investigating improved cryopreservation techniques and lipid alternatives.

**Table 2:** Summary of developed circRNA vaccines.

Vaccine	Antigen	IRES	Cyclization Method	Delivery	Research Team	Period	Reference
circRNA RBD	Encodes SARS-CoV-2 RBD receptor binding domain	CVB3	Type I intron self-splicing	LNP	Therorna	March 2022	[30]
circRNA RBD	Encodes SARS-CoV-2 RBD receptor binding domain	CVB3	Type II intron self-splicing	LNP	Circode Bio	May 2022	[55]
VFLIP-X	Encodes SARS-CoV-2 spiking protein	CVB3	T4 RNA ligase	LNP	Patompon Wongtrakoongate et al. Mahidol University	March 2022	[94]
circRNA^OVA-Luc^	Encodes OVA (257–264) luciferase	CVB3	Type I intron self-splicing	LNP	Lin Xin et al. Tsinghua University School of Medicine	August 2022	[9]
CircRNA encoding cytokines	Encodes IL-15, IL-12, GM-CSF, IFN-α2b	EV29	Clean-PIE	LNP	CureMed	September 2022	[45]
IL12-circRNA	IL-12	CVB3	Type I intron self-splicing	LNP	B. Li, University Health Network Toronto	April 2024	[92]
cirA29L, cirA35R, cirB6R, and cirM1R	MPXV proteins A29L, A35R, B6R, and M1R	/	Type II intron self-splicing	LNP	Circode Bio	April 2024	[95]

### 5.3. Exosome Delivery

Exosomes are extracellular vesicles 40–160 nm in size secreted by cells, and contain various proteins [96], lipids, and nucleic acids that are unique to cells [97]. Exosomes are formed from a lipid bilayer containing various lipids such as cholesterol, diglycerides, sphingomyelin, and monosialotetrahexosyl ganglioside to maintain membrane rigidity and fluidity [98]. Because of their inherent low immunogenicity and biocompatibility, exosomes can overcome various biological barriers to transport proteins, lipids, and genes between different cellular environments. Thus, they are attractive nanocarriers for the delivery of engineered circRNAs [99]. Exosomes can be isolated from many different cell types by ultracentrifugation, microfluidic, polymer precipitation, immunoaffinity capture, and size-exclusion chromatography [100]. Currently, the isolation of exosomes from biological fluids is not only costly, but also low in yield, which limits the use of cell-derived exosomes. Artificial methods for the preparation of exosomes have been proposed [101], such as manufacturing by extrusion, filtration, microfluidics, sonication, foaming, or modifying the membrane of liposomes to mimic the composition and properties of the cell membrane [102]. The obtained exosomes can be loaded with RNA by a variety of methods, including the incubation of exosomes with RNA at a specific temperature, transfection, electroporation, and sonication [103]. Similar to nanoparticles, exosome delivery systems can effectively protect nucleic acid molecules from degradation and promote cellular uptake. Moreover, exosomes, as a natural endogenous carrier system, have the advantages of high biocompatibility, low toxicity, non-immunogenicity, and better permeability than synthetic nanoparticles [104]. COVID-19 immunization by exosome-mediated mRNA delivery has been established [105], and therapeutic endogenous circRNA has been delivered by loading cell-derived exosomes to fulfill their native functions in various disease pathways [106]. Yang et al. first performed a comprehensive analysis of circRNA in the plasma of patients with acute ischemic stroke by using circRNA microarray technology. circSCMH1 was found to be significantly reduced, and the same phenomenon was also found in a mouse model of stroke. To enhance the amount of circSCMH1 in the brain, they used engineered rabies virus glycoprotein (RVG)-modified extracellular vesicles to selectively deliver circSCMH1 to the brains of mice and rhesus monkeys in stroke models [107]. Their results showed that this effectively improved functional recovery. Specific conditions are required for the storage of exosomes. Improper storage or changes in pH or temperature conditions, e.g., via repeated freezing and thawing, may lead to degradation. So, this creates challenges for transportation and storage [108]. In addition, the study of exosome content is not sufficient, and there is still a huge gap in utilizing the exosome system as an ideal drug carrier.

### 5.4. Other Potential Delivery Vectors

CircRNA and mRNA have similar encapsulation properties, and the delivery of circRNA can mimic other delivery strategies used for mRNA [109]. Protamine is a naturally occurring protein that is primarily made up of positively charged L-arginine and is obtained from salmon spermatozoa. It is perfect for stabilizing and concentrating negatively charged mRNA [110]. In mouse models, protamine–mRNA complexes resulted in strong antigen-specific CD4^+^ T-cell, CD8^+^ T-cell, and B-lymphocyte responses, considerably increasing mRNA transfection efficiency [111]. mRNA can be efficiently encapsulated in hydrogels, which are three-dimensional, soft, water-swelling, biodegradable materials. Hydrogels, as RNA delivery carriers, can effectively prevent enzymatic degradation, and the targeted distribution of mRNA to particular cells or tissues is made possible by nanogel technology. Crucially, several existing mRNA vaccines, including those for influenza, VZV, RSV, and SARS-CoV-2, usually need to be administered many times. Hydrogels can achieve pulsed or sustained release, with the potential to achieve single-shot immunization, which could largely improve medication compliance [112]. Controlled-release hydrogels, an alternative to LNP delivery systems, may make vaccination easier, faster, more convenient, and more successful while lessening the strain on healthcare systems.

## 6. Applications of RNA Vaccines

An increasing number of studies demonstrate that RNA therapeutic platforms show satisfactory efficacy in the treatment of diseases, such as viral infectious diseases, tumors, and other autoimmune diseases [113,114,115]. Due to the similarity in the action mechanism of protein expression, it is possible to extend the application of mRNA vaccines to circRNA. At present, laboratory studies of engineered circRNA vaccines are mainly focused on sequence design optimization and disease feasibility. These are still in the preclinical evaluation stage, and no clinical studies exist as of yet. Up to now, the application research on circRNA vaccines is mainly focused on infectious diseases and anti-tumor fields [116]. In the following, we outlined the two therapeutic applications of mRNA vaccines with potential extensions to circRNA.

### 6.1. Vaccines for Infectious Diseases

mRNA vaccines are typically used to tackle infectious diseases by encoding pathogen-specific structural proteins that can be used to prevent pathogenic infections, such as SARS-CoV-2, respiratory syncytial virus (RSV), herpes zoster virus (VZV), cytomegalovirus (CMV), influenza virus (flu), HIV, Zika virus, dengue virus, and monkey pox virus [117]. The COVID-19 outbreak in 2019 brought great health threats to the world, and the mRNA-based COVID-19 vaccines developed by Moderna and BioNTech have achieved more than 95% effectiveness in clinical trials with minimal side effects [49,83,118]. These two vaccines have been approved for emergency use by the U.S. Food and Drug Administration (FDA) and have been widely used worldwide. To date, more than 40 mRNA vaccines for COVID-19 have been tested in clinical trials. In addition, there have been several circRNA vaccines reported to treat SARS-CoV-2 and its emergent variants [30,55,94,119]. Moreover, circRNA vaccines could produce higher RBD antigens compared to the corresponding mRNA vaccines [30,94]. Arevalo et al. developed a nucleoside-modified mRNA-LNP vaccine encoding hemagglutinin antigens from 20 known influenza A and B viruses [120]. This vaccine elicited high levels of cross-reactive and subtype-specific antibodies in mice and ferrets, and reacted against all 20 encoded antigens. IAVIG001 is a recombinant subunit vaccine based on the eOD-GT8 60mer antigen sponsored by the International AIDS Vaccine Initiative (IAVI) that can produce high levels of IgG antibodies against HIV after vaccination. Based on this research, Moderna has collaborated with them to develop mRNA-1644 and mRNA-1644-core, for which phase I clinical trials have been initiated. mRNA-1647 (cytomegalovirus vaccine), mRNA-1345 (RSV vaccine), and mRNA-1010 [121] (seasonal influenza tetravalent vaccine) are undergoing phase III clinical trials. The mRNA-1893 vaccine against the Zika virus has also entered the recruitment stage of phase II clinical trials [117]. Based on the RNActive platform, CureVac company developed the rabies virus mRNA vaccine (CV7201), inducing neutralizing antibodies against the rabies virus in a phase I clinical trial. In addition to viral pathogens, malaria vaccine development is also in the works. Mallory et al. created an antimalarial mRNA vaccine that encodes PfCSP, the immunodominant coat protein of the Plasmodium infestation stage. A booster dose of the vaccine induced prophylaxis against malaria in a mouse model [122]. The latest research reported by Zhou et al. involved the selection of four monkeypox virus (MPXV) antigens (A29L, A35R, B6R, and M1R) and the construction of four circRNA vaccines. The expression of different surface proteins of MPXV can induce neutralizing antibodies and T-cell responses in mice, thereby providing effective protection against vaccinia virus (VACV) [95]. This study demonstrates the potential of circRNA vaccines to prevent monkeypox virus and provides new directions for future vaccine development.

### 6.2. Anti-Tumor Vaccines

mRNA vaccines for cancer immunotherapy have also received widespread attention, and could express defective or modified tumor suppressor proteins and modulate the tumor immune microenvironment. Research is currently focused on using mRNA as a kind of therapeutic vaccine to stimulate and train the immune system to kill cancer cells. By encoding tumor-associated antigens (TAAs) and tumor-specific antigens (TSAs), the immune system is facilitated in recognizing and attacking tumor cells, thereby presenting new opportunities for mRNA products. Currently, more than 20 mRNA vaccines have entered clinical trials for the treatment of tumors. BioNTech’s BNT111 vaccine contains four melanoma-associated antigens (NY-ESO-1, MAGE-A3, tyrosinase, and TPTE). The partial immunization results of the early clinical trial showed that 75% of subjects exhibited specific T-cell responses to at least one TAA. Imaging results from the 42 subjects initially evaluated showed that of the 25 subjects who received only this therapy, 3 experienced partial remission and 7 remained stable, while of the 17 subjects in the vaccine–anti-PD1 combination therapy groups, 6 experienced partial remission [123,124]. BioNTech has developed several vaccine candidates for different cancers [125,126], such as BNT111 and BNT121 for melanoma, BNT112 for prostate cancer, BNT113 for HPV16-positive squamous cell carcinoma of the head and neck, BNT116 for non-small-cell lung cancer, and BNT122 for multiple cancers. BNT121 has induced sustained remissions in melanoma subjects treated with combined checkpoint inhibitors [124]. Up to 20 personalized neoantigens have been encoded in BNT122, which is currently in clinical trials for the treatment of a variety of diseases, including melanoma, colorectal cancer, prostate cancer, and non-small cell lung cancer. The preliminary clinical trial results showed that BNT122 has an acceptable safety profile when administrated alone or in combination with the PD-L1 antibody atezolizumab [123]. The combination of therapeutic cancer vaccines with other immunotherapies, such as immune checkpoint inhibitors, will be a new direction for tumor treatment in the future. Palmer et al. tested a combination of checkpoint inhibitors with a heterologous cancer neoantigen vaccine consisting of an adenovirus vector, ChAd68, and a self-amplifying mRNA vector [127]. They found that this vaccine could generate neoantigen-specific CD8^+^ T-cell responses in non-human primates and subjects in the clinical trial. Recently, Wang et al. developed a novel neoantigen vaccine based on circRNA that induces a potent antitumor immune response [43]. This engineered cirRNA vaccine could express hepatocellular carcinoma-specific tumor neoantigens (Ptpn2_I383T) and elicit a robust T-cell immune response to inhibit the growth of tumors. CircRNA vaccines offer new options and application prospects for immunotherapy in solid tumors. In addition, endosomal circRNA mimics synthesized in vitro can also be used to treat diseases. Huang et al. identified cryptic antigenic peptides bound to human leukocyte antigen class I (HLA- I) in human breast cancer samples [128]. The cryptic antigenic peptides were noncanonically translated by the tumor-specific circRNA circFAM53B. The administration of the vaccine composed of tumor-specific circFAM53B or its coding cryptic peptides in mice with breast cancer tumors or melanoma induced the enhanced infiltration of tumor antigen-specific cytotoxic T cells, thereby effectively controlling tumors. This suggests that vaccination by tumor-specific circRNA could be used as an immunotherapy strategy against malignant tumors.

## 7. Conclusions and Outlook

Engineered circRNAs have become a hotspot in vaccine research due to their high stability, low immunogenicity, and high translation efficiency. Many biopharmaceutical companies and research teams are accelerating the development of circRNA vaccines. Current circRNA synthesis technologies still have limitations, such as low cyclization yields and high costs of reagents (e.g., enzymes). In addition, production strategies and corresponding equipment are not yet mature enough for large-scale production. CircRNA generated in vitro has numerous biological activities, which can lead to cross-reaction and a host of significant adverse effects. Therefore, the sequences of engineered circRNA need to be precise and thoroughly evaluated for biosafety and effectiveness. Due to many problems in research and development, production, quality control, and safety, circRNA research is still in the preclinical stage. In general, there are five main issues that need to be addressed going forward: designing circRNA with high antigenic yields and low immunogenicity; enhancing the cyclization efficiency of linear RNA precursors; sufficiently purifying to prevent contamination; developing appropriate delivery systems; and realizing disease therapeutic applications. New technologies based on circRNA will open up new directions for the treatment and prevention of diseases. circRNA vaccines are expected to be used extensively in clinical studies to inhibit malignant tumors and prevent infectious illnesses.

## Figures and Tables

**Table 1:** Advantages and disadvantages of various vaccines.

Type of Vaccine	Synthetic Long-Peptide Vaccine	DNA Vaccine	mRNA Vaccine	circRNA Vaccine
Synthesis	Synthetic long peptides	Plasmid DNA encoding the antigen	mRNA transcribed in vitro	CircRNA constructed in vitro
Advantages	Stable Safe Easy to purify Long immunization time	Stable (can be stored at room temperature) Simple production process Low cost Mass production	Fast production Low cost Cytoplasmic translation No risk of genomic integration Self-adjuvant effect	More stable and easy to store Cytoplasmic translation No risk of genomic integration Self-adjuvant effect High antigen expression efficiency
Disadvantages	Complex in vitro synthesis High cost Low immune response Potential adverse effects	Risk of genomic integration Must cross the nuclear membrane to be transcribed Carcinogenic risk	Poor stability Easily degradable Harsh storage conditions High risk of side effects	Currently not available for mass production Safety unknown
Improvement program	Use immune adjuvants	Vector and promoter optimization Improve inoculation route	Improvement of target gene expression Improve delivery system	Nucleotide modifications Improve cyclization efficiency Improve purification methods
Reference	[33,34,35]	[36,37]	[38,39,40]	[28,41,42,43]

## References

[1] Zhang C., Zhang B.L. (2023). RNA therapeutics: Updates and future potential. Sci. China-Life Sci..

[2] Sanger H.L., Klotz G., Riesner D., Gross H.J., Kleinschmidt A.K. (1976). Viroids are single-stranded covalently closed circular RNA molecules existing as highly base-paired rod-like structures. Proc. Natl. Acad. Sci. USA.

[3] Patop I.L., Wüst S., Kadener S. (2019). Past, present, and future of circRNAs. Embo J..

[4] Jiang J., Shi S., Zhang W., Li C., Sun L., Ge Q., Li X. (2024). Circ_RPPH1 facilitates progression of breast cancer via miR-1296-5p/TRIM14 axis. Cancer Biol. Ther..

[5] Li F., Zhang L., Li W., Deng J., Zheng J., An M., Lu J., Zhou Y. (2015). Circular RNA ITCH has inhibitory effect on ESCC by suppressing the Wnt/β-catenin pathway. Oncotarget.

[6] Li Q., Zhang Y., Jin P., Chen Y., Zhang C., Geng X., Mun K.S., Phang K.C. (2024). New insights into the potential of exosomal circular RNAs in mediating cancer chemotherapy resistance and their clinical applications. Biomed. Pharmacother..

[7] Yang Y., Fan X.J., Mao M.W., Song X.W., Wu P., Zhang Y., Jin Y.F., Yang Y., Chen L.L., Wang Y. (2017). Extensive translation of circular RNAs driven by N6-methyladenosine. Cell Res..

[8] Wesselhoeft R.A., Kowalski P.S., Anderson D.G. (2018). Engineering circular RNA for potent and stable translation in eukaryotic cells. Nat. Commun..

[9] Li H.J., Peng K., Yang K., Ma W.B., Qi S.L., Yu X.Y., He J., Lin X., Yu G.C. (2022). Circular RNA cancer vaccines drive immunity in hard-to-treat malignancies. Theranostics.

[10] Ju J., Song Y.N., Chen X.Z., Wang T., Liu C.Y., Wang K. (2022). circRNA is a potential target for cardiovascular diseases treatment. Mol. Cell. Biochem..

[11] Kristensen L.S., Andersen M.S., Stagsted L.V.W., Ebbesen K.K., Hansen T.B., Kjems J. (2019). The biogenesis, biology and characterization of circular RNAs. Nat. Rev. Genet..

[12] Rahmati Y., Asemani Y., Aghamiri S., Ezzatifar F., Najafi S. (2021). CiRS-7/CDR1as; An oncogenic circular RNA as a potential cancer biomarker. Pathol. Res. Pract..

[13] Guo Z.Y., Cao Q.D., Zhao Z., Song C.L. (2020). Biogenesis, Features, Functions, and Disease Relationships of a Specific Circular RNA: CDR1as. Aging Dis..

[14] Hu Z.Y., Chen J.R., Wang M.L., Weng W.Z., Chen Y., Pan Y.F. (2022). Rheumatoid arthritis fibroblast-like synoviocytes maintain tumor-like biological characteristics through ciRS-7-dependent regulation of miR-7. Mol. Biol. Rep..

[15] Lei M., Zheng G.T., Ning Q.Q., Zheng J.N., Dong D. (2020). Translation and functional roles of circular RNAs in human cancer. Mol. Cancer.

[16] Zheng W., Wang L., Geng S., Yang L., Lv X., Xin S., Xu T. (2023). CircMIB2 therapy can effectively treat pathogenic infection by encoding a novel protein. Cell Death Dis..

[17] Wang Y., Wang Z.F. (2015). Efficient backsplicing produces translatable circular mRNAs. Rna.

[18] Chen C.Y., Sarnow P. (1995). Initiation of protein synthesis by the eukaryotic translational apparatus on circular RNAs. Science.

[19] Zhang L.L., Hou C.F., Chen C., Guo Y.X., Yuan W.T., Yin D.T., Liu J.B., Sun Z.Q. (2020). The role of N6-methyladenosine (m6A) modification in the regulation of circRNAs. Mol. Cancer.

[20] Duan J.L., Chen W., Xie J.J., Zhang M.L., Nie R.C., Liang H., Mei J., Han K., Xiang Z.C., Wang F.W. (2022). A novel peptide encoded by N6-methyladenosine modified circMAP3K4 prevents apoptosis in hepatocellular carcinoma. Mol. Cancer.

[21] Chen C.K., Cheng R., Demeter J., Chen J., Weingarten-Gabbay S., Jiang L., Snyder M.P., Weissman J.S., Segal E., Jackson P.K. (2021). Structured elements drive extensive circular RNA translation. Mol. Cell.

[22] Legnini I., Di Timoteo G., Rossi F., Morlando M., Briganti F., Sthandier O., Fatica A., Santini T., Andronache A., Wade M. (2017). Circ-ZNF609 Is a Circular RNA that Can Be Translated and Functions in Myogenesis. Mol. Cell.

[23] Shang Q.F., Du H.Z., Wu X.W., Guo Q., Zhang F.H., Gong Z.Q., Jiao T., Guo J., Kong Y. (2022). FMRP ligand circZNF609 destabilizes RAC1 mRNA to reduce metastasis in acral melanoma and cutaneous melanoma. J. Exp. Clin. Cancer Res..

[24] Wang K.Q., Ye M.L., Qiao X., Yu Z.W., Wu C.X., Zheng J.F. (2022). Circular RNA Fibroblast Growth Factor Receptor 1 Promotes Pancreatic Cancer Progression by Targeting MicroRNA-532-3p/PIK3CB Axis. Pancreas.

[25] Abe N., Matsumoto K., Nishihara M., Nakano Y., Shibata A., Maruyama H., Shuto S., Matsuda A., Yoshida M., Ito Y. (2015). Rolling Circle Translation of Circular RNA in Living Human Cells. Sci. Rep..

[26] Hobernik D., Bros M. (2018). DNA Vaccines-How Far From Clinical Use?. Int. J. Mol. Sci..

[27] Ju M., Kim D., Son G., Han J. (2023). Circular RNAs in and out of Cells: Therapeutic Usages of Circular RNAs. Mol. Cells.

[28] Chen R., Wang S.K., Belk J.A., Amaya L., Li Z.J., Cardenas A., Abe B.T., Chen C.K., Wender P.A., Chang H.Y. (2023). Engineering circular RNA for enhanced protein production. Nat. Biotechnol..

[29] Costello A., Lao N.T., Barron N., Clynes M. (2019). Continuous translation of circularized mRNA improves recombinant protein titer. Metab. Eng..

[30] Qu L., Yi Z., Shen Y., Lin L., Chen F., Xu Y., Wu Z., Tang H., Zhang X., Tian F. (2022). Circular RNA vaccines against SARS-CoV-2 and emerging variants. Cell.

[31] Chen Y.G., Kim M.V., Chen X., Batista P.J., Aoyama S., Wilusz J.E., Iwasaki A., Chang H.Y. (2017). Sensing Self and Foreign Circular RNAs by Intron Identity. Mol. Cell.

[32] Ren Y., Manoharan T., Liu B.J., Cheng C.Z.M., Siew B.E., Cheong W.K., Lee K.Y., Tan I.J.W., Lieske B., Tan K.K. (2024). Circular RNA as a source of neoantigens for cancer vaccines. J. Immunother. Cancer.

[33] Hu Z.T., Leet D.E., Allesoe R.L., Oliveira G., Li S.Q., Luoma A.M., Liu J.Y., Forman J., Huang T., Iorgulescu J.B. (2021). Personal neoantigen vaccines induce persistent memory T cell responses and epitope spreading in patients with melanoma. Nat. Med..

[34] Mork S.K., Kadivar M., Bol K.F., Draghi A., Westergaard M.C.W., Skadborg S.K., Overgaard N., Sorensen A.B., Rasmussen I.S., Andreasen L.V. (2022). Personalized therapy with peptide-based neoantigen vaccine (EVX-01) including a novel adjuvant, CAF^®®^09b, in patients with metastatic melanoma. Oncoimmunology.

[35] Rabu C., Rangan L., Florenceau L., Fortu A., Charpentier M., Dupré E., Paolini L., Beauvillain C., Dupel E., Latouche J.B. (2019). Cancer vaccines: Designing artificial synthetic long peptides to improve presentation of class I and class II T cell epitopes by dendritic cells. Oncoimmunology.

[36] Lv Z., Zhang X., Zhao K., Du L., Wang X., Chu Y., Huang T. (2024). Co-immunization with DNA vaccines encoding yidR and IL-17 augments host immune response against Klebsiella pneumoniae infection in mouse model. Virulence.

[37] Zhu L.F., Cui X.J., Yan Z.L., Tao Y.F., Shi L., Zhang X.W., Yao Y.F., Shi L. (2024). Design and evaluation of a multi-epitope DNA vaccine against HPV16. Hum. Vaccines Immunother..

[38] Jaber H.M., Ebdah S., Mahmoud S.A.A., Abu-Qatouseh L., Jaber Y.H. (2024). Comparison of T cells mediated immunity and side effects of mRNA vaccine and conventional COVID-19 vaccines administrated in Jordan. Hum. Vaccines Immunother..

[39] Shi R.M., Liu X.L., Wang Y.J., Pan M.L., Wang S.Q., Shi L., Ni B.B. (2024). Long-term stability and immunogenicity of lipid nanoparticle COVID-19 mRNA vaccine is affected by particle size. Hum. Vaccines Immunother..

[40] Yao R.H., Xie C.Y., Xia X.J. (2024). Recent progress in mRNA cancer vaccines. Hum. Vaccines Immunother..

[41] Chen X.J., Wang C., Lu Y. (2023). Tactics targeting circular mRNA biosynthesis. Biotechnol. Bioeng..

[42] Wan J.W., Wang Z.M., Wang L.L., Wu L.Q., Zhang C.G., Zhou M., Fu Z.F., Zhao L. (2024). Circular RNA vaccines with long-term lymph node-targeting delivery stability after lyophilization induce potent and persistent immune responses. Mbio.

[43] Wang F., Cai G., Wang Y., Zhuang Q., Cai Z., Li Y., Gao S., Li F., Zhang C., Zhao B. (2024). Circular RNA-based neoantigen vaccine for hepatocellular carcinoma immunotherapy. MedComm.

[44] Amaya L., Grigoryan L., Li Z.J., Lee A., Wender P.A., Pulendran B., Chang H.Y. (2023). Circular RNA vaccine induces potent T cell responses. Proc. Natl. Acad. Sci. USA.

[45] Yang J.L., Zhu J.F., Sun J.J., Chen Y.Y., Du Y.R., Tan Y.L., Wu L.P., Zhai M.T., Wei L.X., Li N. (2022). Intratumoral delivered novel circular mRNA encoding cytokines for immune modulation and cancer therapy. Mol. Ther. Nucleic Acids.

[46] Schaeffer D., Tsanova B., Barbas A., Reis F.P., Dastidar E.G., Sanchez-Rotunno M., Arraiano C.M., van Hoof A. (2009). The exosome contains domains with specific endoribonuclease, exoribonuclease and cytoplasmic mRNA decay activities. Nat. Struct. Mol. Biol..

[47] Zhou M., Xiao M.S., Li Z.G., Huang C. (2021). New progresses of circular RNA biology: From nuclear export to degradation. Rna Biol..

[48] Hansen T.B., Jensen T.I., Clausen B.H., Bramsen J.B., Finsen B., Damgaard C.K., Kjems J. (2013). Natural RNA circles function as efficient microRNA sponges. Nature.

[49] Vergnes J.N. (2021). Safety and Efficacy of the BNT162b2 mRNA COVID-19 Vaccine. N. Engl. J. Med..

[50] Vishweshwaraiah Y.L., Dokholyan N.V. (2022). mRNA vaccines for cancer immunotherapy. Front. Immunol..

[51] Liu J., Guo C., Fu J., Liu D., Liu G., Sun B., Deng M., Guo Y., Li Y. (2024). Identification and Functional Analysis of circRNAs during Goat Follicular Development. Int. J. Mol. Sci..

[52] Sharma N.K., Dwivedi P., Bhushan R., Maurya P.K., Kumar A., Dakal T.C. (2024). Engineering circular RNA for molecular and metabolic reprogramming. Funct. Integr. Genom..

[53] Su C.I., Chuang Z.S., Shie C.T., Wang H.I., Kao Y.T., Yu C.Y. (2024). A cis-acting ligase ribozyme generates circular RNA in vitro for ectopic protein functioning. Nat. Commun..

[54] Unti M.J., Jaffrey S.R. (2024). Highly efficient cellular expression of circular mRNA enables prolonged protein expression. Cell Chem. Biol..

[55] Chen C., Wei H., Zhang K., Li Z., Wei T., Tang C., Yang Y., Wang Z.J.B. (2022). A flexible, efficient, and scalable platform to produce circular RNAs as new therapeutics. BioRxiv.

[56] Jackson R.J., Hellen C.U.T., Pestova T.V. (2010). The mechanism of eukaryotic translation initiation and principles of its regulation. Nat. Rev. Mol. Cell Biol..

[57] Rong M., He B., McAllister W.T., Durbin R.K. (1998). Promoter specificity determinants of T7 RNA polymerase. Proc. Natl. Acad. Sci. USA.

[58] Usman N., Cedergren R. (1992). Exploiting the chemical synthesis of RNA. Trends Biochem. Sci..

[59] Obi P., Chen Y.G. (2021). The design and synthesis of circular RNAs. Methods.

[60] He W., Zhang X.Y., Zou Y.X.Y., Li J., Chang L., He Y.C., Jin Q.H., Ye J.R. (2024). Effective synthesis of circRNA via a thermostable T7 RNA polymerase variant as the catalyst. Front. Bioeng. Biotechnol..

[61] Chen X., Lu Y. (2021). Circular RNA: Biosynthesis in vitro. Front. Bioeng. Biotechnol..

[62] Bai Y., Liu D., He Q., Liu J.Y., Mao Q.Y., Liang Z.L. (2023). Research progress on circular RNA vaccines. Front. Immunol..

[63] Lee K.H., Kim S., Lee S.W. (2022). Pros and Cons of In Vitro Methods for Circular RNA Preparation. Int. J. Mol. Sci..

[64] Costello A., Lao N.T., Barron N., Clynes M. (2020). Reinventing the Wheel: Synthetic Circular RNAs for Mammalian Cell Engineering. Trends Biotechnol..

[65] Breuer J., Rossbach O. (2020). Production and Purification of Artificial Circular RNA Sponges for Application in Molecular Biology and Medicine. Methods Protoc..

[66] Carmona E.M. (2019). Circular RNA: Design Criteria for Optimal Therapeutical Utility.

[67] Puttaraju M., Been M.D. (1992). Group I permuted intron-exon (PIE) sequences self-splice to produce circular exons. Nucleic Acids Res..

[68] Ford E., Ares M. (1994). Synthesis of circular RNA in bacteria and yeast using RNA cyclase ribozymes derived from a group I intron of phage T4. Proc. Natl. Acad. Sci. USA.

[69] Liu C.X., Guo S.K., Nan F., Xu Y.F., Yang L., Chen L.L. (2022). RNA circles with minimized immunogenicity as potent PKR inhibitors. Mol. Cell.

[70] Qiu Z., Zhao Y., Hou Q., Zhu J., Zhai M., Li D., Li Y., Liu C., Li N., Cao Y.J.b. (2022). Clean-PIE: A novel strategy for efficiently constructing precise circRNA with thoroughly minimized immunogenicity to direct potent and durable protein expression. BioRxiv.

[71] Zhang Y., Yang L., Chen L.L. (2016). Characterization of circular RNAs. Long Non Coding RNAs Methods Protocols.

[72] Zhang N.N., Li X.F., Deng Y.Q., Zhao H., Huang Y.J., Yang G., Huang W.J., Gao P., Zhou C., Zhang R.R. (2020). A Thermostable mRNA Vaccine against COVID-19. Cell.

[73] Niu M.T., Wang C.Y., Chen Y.J., Zou Q., Xu L. (2024). Identification, characterization and expression analysis of circRNA encoded by SARS-CoV-1 and SARS-CoV-2. Brief. Bioinform..

[74] Li M.Y., Li Y., Li S.Q., Jia L., Wang H.M., Li M., Deng J., Zhu A.L., Ma L.Q., Li W.H. (2022). The nano delivery systems and applications of mRNA. Eur. J. Med. Chem..

[75] Paunovska K., Loughrey D., Dahlman J.E. (2022). Drug delivery systems for RNA therapeutics. Nat. Rev. Genet..

[76] Li Y.D., Chi W.Y., Su J.H., Ferrall L., Hung C.F., Wu T.C. (2020). Coronavirus vaccine development: From SARS and MERS to COVID-19. J. Biomed. Sci..

[77] Sun X., Zeng L., Huang Y. (2019). Transcutaneous delivery of DNA/mRNA for cancer therapeutic vaccination. J. Gene Med..

[78] Husseini R.A., Abe N., Hara T., Abe H., Kogure K. (2023). Use of Iontophoresis Technology for Transdermal Delivery of a Minimal mRNA Vaccine as a Potential Melanoma Therapeutic. Biol. Pharm. Bull..

[79] Gregoriadis G. (2016). Liposomes in Drug Delivery: How It All Happened. Pharmaceutics.

[80] Harashima H., Sakata K., Funato K., Kiwada H. (1994). Enhanced hepatic uptake of liposomes through complement activation depending on the size of liposomes. Pharm. Res..

[81] Chaudhary N., Weissman D., Whitehead K.A. (2021). mRNA vaccines for infectious diseases: Principles, delivery and clinical translation. Nat. Rev. Drug Discov..

[82] Polack F.P., Thomas S.J., Kitchin N., Absalon J., Gurtman A., Lockhart S., Perez J.L., Marc G.P., Moreira E.D., Zerbini C. (2020). Safety and Efficacy of the BNT162b2 mRNA COVID-19 Vaccine. N. Engl. J. Med..

[83] Baden L.R., El Sahly H.M., Essink B., Kotloff K., Frey S., Novak R., Diemert D., Spector S.A., Rouphael N., Creech C.B. (2021). Efficacy and Safety of the mRNA-1273 SARS-CoV-2 Vaccine. N. Engl. J. Med..

[84] Hou X.C., Zaks T., Langer R., Dong Y.Z. (2021). Lipid nanoparticles for mRNA delivery. Nat. Rev. Mater..

[85] Han X.X., Zhang H.W., Butowska K., Swingle K.L., Alameh M.G., Weissman D., Mitchell M.J. (2021). An ionizable lipid toolbox for RNA delivery. Nat. Commun..

[86] Nitika, Wei J., Hui A.M. (2022). The Delivery of mRNA Vaccines for Therapeutics. Life.

[87] Li Y., Fang H.T., Zhang T., Wang Y., Qi T.T., Li B., Jiao H.P. (2022). Lipid-mRNA nanoparticles landscape for cancer therapy. Front. Bioeng. Biotechnol..

[88] Eygeris Y., Gupta M., Kim J., Sahay G. (2022). Chemistry of Lipid Nanoparticles for RNA Delivery. Acc. Chem. Res..

[89] Ball R.L., Hajj K.A., Vizelman J., Bajaj P., Whitehead K.A. (2018). Lipid Nanoparticle Formulations for Enhanced Co-delivery of siRNA and mRNA. Nano Lett..

[90] Cullis P.R., Hope M.J. (2017). Lipid Nanoparticle Systems for Enabling Gene Therapies. Mol. Ther..

[91] Wesselhoeft R.A., Kowalski P.S., Parker-Hale F.C., Huang Y.X., Bisaria N., Anderson D.G. (2019). RNA Circularization Diminishes Immunogenicity and Can Extend Translation Duration In Vivo. Mol. Cell.

[92] Xu S.F., Xu Y., Solek N.C., Chen J.A., Gong F.L., Varley A.J., Golubovic A., Pan A.N., Dong S.T., Zheng G. (2024). Tumor-Tailored Ionizable Lipid Nanoparticles Facilitate IL-12 Circular RNA Delivery for Enhanced Lung Cancer Immunotherapy. Adv. Mater..

[93] Ndeupen S., Qin Z., Jacobsen S., Bouteau A., Estanbouli H., Igyártó B.Z. (2021). The mRNA-LNP platform’s lipid nanoparticle component used in preclinical vaccine studies is highly inflammatory. Iscience.

[94] Seephetdee C., Bhukhai K., Buasri N., Leelukkanaveera P., Lerdwattanasombat P., Manopwisedjaroen S., Phueakphud N., Kuhaudomlarp S., Olmedillas E., Saphire E.O. (2022). A circular mRNA vaccine prototype producing VFLIP-X spike confers a broad neutralization of SARS-CoV-2 variants by mouse sera. Antivir. Res..

[95] Zhou J., Ye T., Yang Y., Li E., Zhang K., Wang Y., Chen S., Hu J., Zhang K., Liu F. (2024). Circular RNA vaccines against monkeypox virus provide potent protection against vaccinia virus infection in mice. Mol. Ther. J. Am. Soc. Gene Ther..

[96] Zhou Q., Fang L., Tang Y.C., Wang Q., Tang X., Zhu L.X., Peng N., Wang B.Y., Song W.K., Fu H. (2024). Exosome-mediated delivery of artificial circular RNAs for gene therapy of bladder cancer. J. Cancer.

[97] Moon B., Chang S. (2022). Exosome as a Delivery Vehicle for Cancer Therapy. Cells.

[98] Kalluri R., LeBleu V.S. (2020). The biology, function, and biomedical applications of exosomes. Science.

[99] Amiri A., Bagherifar R., Dezfouli E.A., Kiaie S.H., Jafari R., Ramezani R. (2022). Exosomes as bio-inspired nanocarriers for RNA delivery: Preparation and applications. J. Transl. Med..

[100] Sun Y.F., Pi J., Xu J.F. (2021). Emerging Role of Exosomes in Tuberculosis: From Immunity Regulations to Vaccine and Immunotherapy. Front. Immunol..

[101] Li Y.J., Wu J.Y., Liu J.H., Xu W.J., Qiu X.H., Huang S., Hu X.B., Xiang D.X. (2021). Artificial exosomes for translational nanomedicine. J. Nanobiotechnol..

[102] Wang X.D., Zhao X., Zhong Y.X., Shen J.H., An W.L. (2022). Biomimetic Exosomes: A New Generation of Drug Delivery System. Front. Bioeng. Biotechnol..

[103] Lu Y.C., Huang W., Li M., Zheng A.P. (2023). Exosome-Based Carrier for RNA Delivery: Progress and Challenges. Pharmaceutics.

[104] Du W.W., Fang L., Yang W.N., Wu N., Awan F.M., Yang Z.G., Yang B.B. (2017). Induction of tumor apoptosis through a circular RNA enhancing Foxo3 activity. Cell Death Differ..

[105] Tsai S.J., Guo C., Sedgwick A., Kanagavelu S., Nice J., Shetty S., Landaverde C., Atai N.A., Gould S.J. (2020). Exosome-mediated mRNA delivery for SARS-CoV-2 vaccination. BioRxiv.

[106] Wang X.Y., Zhang H.Y., Yang H.O., Bai M., Ning T., Deng T., Liu R., Fan Q., Zhu K.G., Li J.L. (2020). Exosome-delivered circRNA promotes glycolysis to induce chemoresistance through the miR-122-PKM2 axis in colorectal cancer. Mol. Oncol..

[107] Yang L., Han B., Zhang Z.T., Wang S.G., Bai Y., Zhang Y., Tang Y., Du L.L., Xu L., Wu F.F. (2020). Extracellular Vesicle-Mediated Delivery of Circular RNA SCMH1 Promotes Functional Recovery in Rodent and Nonhuman Primate Ischemic Stroke Models. Circulation.

[108] Cheng Y.R., Zeng Q.Y., Han Q., Xia W.L. (2019). Effect of pH, temperature and freezing-thawing on quantity changes and cellular uptake of exosomes. Protein Cell.

[109] Loan Young T., Chang Wang K., James Varley A., Li B. (2023). Clinical delivery of circular RNA: Lessons learned from RNA drug development. Adv. Drug Deliv. Rev..

[110] Jarzebska N.T., Mellett M., Frei J., Kundig T.M., Pascolo S. (2021). Protamine-Based Strategies for RNA Transfection. Pharmaceutics.

[111] Fotin-Mleczek M., Duchardt K.M., Lorenz C., Pfeiffer R., Ojkic-Zrna S., Probst J., Kallen K.J. (2011). Messenger RNA-based Vaccines With Dual Activity Induce Balanced TLR-7 Dependent Adaptive Immune Responses and Provide Antitumor Activity. J. Immunother..

[112] Zhong R.B., Talebian S., Mendes B.B., Wallace G., Langer R., Conde J., Shi J.J. (2023). Hydrogels for RNA delivery. Nat. Mater..

[113] Flemming A. (2021). mRNA vaccine shows promise in autoimmunity. Nat. Rev. Immunol..

[114] Guo X.R., Wang X.L., Li M.C., Yuan Y.H., Chen Y., Zou D.D., Bian L.J., Li D.S. (2015). PDX-1 mRNA-induced reprogramming of mouse pancreas-derived mesenchymal stem cells into insulin-producing cells in vitro. Clin. Exp. Med..

[115] Krienke C., Kolb L., Diken E., Streuber M., Kirchhoff S., Bukur T., Akilli-Öztürk Ö., Kranz L.M., Berger H., Petschenka J. (2021). A noninflammatory mRNA vaccine for treatment of experimental autoimmune encephalomyelitis. Science.

[116] Niu D., Wu Y.R., Lian J.Q. (2023). Circular RNA vaccine in disease prevention and treatment. Signal Transduct. Target. Ther..

[117] Essink B., Chu L.R.C., Seger W., Barranco E., Le Cam N., Bennett H., Faughnan V., Pajon R., Paila Y., Bollman B. (2023). The safety and immunogenicity of two Zika virus mRNA vaccine candidates in healthy flavivirus baseline seropositive and seronegative adults: The results of two randomised, placebo-controlled, dose-ranging, phase 1 clinical trials. Lancet Infect. Dis..

[118] Chalkias S., Harper C., Vrbicky K., Walsh S.R., Essink B., Brosz A., McGhee N., Tomassini J.E., Chen X., Chang Y. (2022). A Bivalent Omicron-Containing Booster Vaccine against COVID-19. N Engl. J. Med..

[119] Huang K., Li N., Li Y., Zhu J., Fan Q., Yang J., Gao Y., Liu Y., Hou Q., Gao S. (2022). Delivery of Circular mRNA via Degradable Lipid Nanoparticles against SARS-CoV-2 Delta Variant. BioRxiv.

[120] Arevalo C.P., Bolton M.J., Le Sage V., Ye N.Q., Furey C., Muramatsu H., Alameh M.G., Pardi N., Drapeau E.M., Parkhouse K. (2022). A multivalent nucleoside-modified mRNA vaccine against all known influenza virus subtypes. Science.

[121] Lee I.T., Nachbagauer R., Ensz D., Schwartz H., Carmona L., Schaefers K., Avanesov A., Stadlbauer D., Henry C., Chen R. (2023). Safety and immunogenicity of a phase 1/2 randomized clinical trial of a quadrivalent, mRNA-based seasonal influenza vaccine (mRNA-1010) in healthy adults: Interim analysis. Nat. Commun..

[122] Mallory K.L., Taylor J.A., Zou X.Y., Waghela I.N., Schneider C.G., Sibilo M.Q., Punde N.M., Perazzo L.C., Savransky T., Sedegah M. (2021). Messenger RNA expressing PfCSP induces functional, protective immune responses against malaria in mice. Npj Vaccines.

[123] Barbier A.J., Jiang A.Y.J., Zhang P., Wooster R., Anderson D.G. (2022). The clinical progress of mRNA vaccines and immunotherapies. Nat. Biotechnol..

[124] Sahin U., Oehm P., Derhovanessian E., Jabulowsky R.A., Vormehr M., Gold M., Maurus D., Schwarck-Kokarakis D., Kuhn A.N., Omokoko T. (2020). An RNA vaccine drives immunity in checkpoint-inhibitor-treated melanoma. Nature.

[125] He K., McKean M., Balaraman R., Shah S., Arrowsmith E., Peguero J., Hamm J., He A., Spira A.I., Joshi R. (2023). 599 Single-agent safety and activities of target-preserving anti-CTLA-4 antibody gotistobart (ONC-392/BNT316) in PD-(L) 1 resistant metastatic NSCLC and population PK analysis in patients with solid tumors. BMJ Spec. J..

[126] Moore K.N., Sabanathan D., Du Y.Q., Duan H.X., Li X.M., Wang F., Marathe O., Yang H., Makker V., Growdon W. (2023). Safety and efficacy of DB-1303 in patients with advanced/metastatic solid tumors: A multicenter, open-label, first-in-human, phase 1/2a study. J. Clin. Oncol..

[127] Palmer C.D., Rappaport A.R., Davis M.J., Hart M.G., Scallan C.D., Hong S.J., Gitlin L., Kraemer L.D., Kounlavouth S., Yang A.R. (2022). Individualized, heterologous chimpanzee adenovirus and self-amplifying mRNA neoantigen vaccine for advanced metastatic solid tumors: Phase 1 trial interim results. Nat. Med..

[128] Huang D., Zhu X., Ye S., Zhang J., Liao J., Zhang N., Zeng X., Wang J., Yang B., Zhang Y. (2024). Tumour circular RNAs elicit anti-tumour immunity by encoding cryptic peptides. Nature.

